# Esophageal motility disorders in symptomatic patients and its relation to age

**DOI:** 10.1186/s12876-023-02709-3

**Published:** 2023-03-11

**Authors:** Ahmed Abdelghani, Alhosaeyn Ibrahim, El-Sayed El-Sayed, Mohammad El Sherbiny, Ahmed Al-Badry

**Affiliations:** Internal Medicine Department, Faculty of Medicine, Cairo University, Cairo, Egypt

**Keywords:** Esophagus, Elderly, Dysfunction, Motility

## Abstract

**Background:**

Although swallowing disorders can impact individuals of any age, some are unique to the elderly, and others are frequent. Some disorders, such as achalasia, are diagnosed by esophageal manometry studies, which assess lower esophageal sphincter (LES) pressure and relaxation, peristalsis in the esophageal body, and contraction wave characteristics. This research aimed to evaluate esophageal motility dysfunction in symptomatic patients and its relation to age.

**Methods:**

Conventional esophageal manometry was performed on 385 symptomatic patients who were divided into two groups; Group A (age < 65 years old) and Group B (age ≥ 65 years). The geriatric assessment for Group B included cognitive, functional, and clinical frailty scales (CFS). Additionally, a nutritional assessment was done for all patients.

**Results:**

About one-third of the patients (33%) had achalasia, in which manometric results were significantly higher in Group B (43.4%) than in Group A (28.7%) (*P* = 0.016).

The resting lower esophageal sphincter (LES) pressure, as determined by manometry examination, was significantly lower in Group A than in Group B. In contrast, complete LES relaxation percentage and normal esophageal body peristalsis were significantly higher in Group A than in Group B. Patients who exhibited evidence of achalasia in the manometric study had a significantly increased risk of established malnutrition and functional impairment.

**Conclusions:**

Achalasia is a prevalent cause of dysphagia in elderly patients, placing them at risk of malnutrition and functional impairment. Thus, a multidisciplinary approach is vital when providing care for this population.

**Supplementary Information:**

The online version contains supplementary material available at 10.1186/s12876-023-02709-3.

## Introduction

Aging is primarily characterized by a progressive loss of physiological integrity, resulting in diminished function and increased susceptibility to death. The elderly population is currently defined as people aged 65 years or above. With older adults comprising 7.3% of the population in 1991, there has been a global trend toward a demographic increase in individuals aged 60 years and older. By 2050, the percentage will rise to 22% (two billion people) [1].

Studies have shown that aberrant esophageal motility is more common with age. Healthy adults over 75 have exhibited decreased basal lower esophageal sphincter (LES) tone and an increased mean integrated relaxation pressure [2].

Although most upper gastrointestinal symptoms are attributed to age-related diseases and the medications used for their treatment, age-related structural and functional changes lead to esophageal motility abnormalities [3]. In nursing homes, dysphagia rates among senior patients can reach 70%. Dysphagia significantly impacts patients’ quality of life, leading to malnutrition, worsening their overall health and increasing their vulnerability [4]. Bolus production and transmission might be impacted by xerostomia (dry mouth), poorly managed diabetes, medications, such as anticholinergics [5], and neurologic conditions, such as cerebral vascular accidents [6]. Research has suggested that in aging individuals, a functional decrease in the percentage of swallows accompanied by complete LES relaxation may occur, resulting in ineffective esophageal motility, even if the overall esophageal function is intact [7].

Achalasia is uncommon, affecting 1 to 2 individuals per 100,000 people worldwide, with a prevalence of 1 in 10,000 [8]. The actual etiology of achalasia is uncertain, with a secondary peak in individuals over 65. However, it is more prevalent in individuals between the ages of 20 and 40 [9]. End-stage achalasia is characterized by significant esophageal tortuosity and dilation (> 6 cm), increasing the risk of malnutrition, aspiration pneumonia, and squamous esophageal carcinoma [10].

Geriatric assessment can refer to a simple or more intense multidisciplinary approach known as a comprehensive geriatric assessment (CGA) to assess an older person’s physical and mental health, functional ability, social situation [11], and distinguishing clinical Alzheimer-type dementia from mild cognitive impairment [12]. Research has shown that frail older adults have limited functional reserves, which can lead to immobility, confusion, and malnutrition [13]. We aimed to evaluate the effect of aging on manometric results in different esophageal motility disorders.

## Patients and methods

This prospective cross-sectional analytical study included 385 patients with upper GIT symptoms of dysphagia, heartburn, or noncardiac chest pain. All patients were subjected to a recent upper endoscopy. They were referred for a conventional esophageal manometry study at the gastroenterology motility unit from December 2019 to June 2022. The current study enrolled 194 males and 191 females. Patients were divided into two groups according to the World Health Organization age criterion for distinguishing the older adult from the younger adult [14] into two groups; Group A (< 65 years old) and Group B (≥ 65 years old).

Patients older than 18 who complained of heartburn, regurgitation, dysphagia, or noncardiac chest pain without any endoscopic evidence of oropharyngeal or esophageal strictures, rings, erosions, ulcers, or benign and malignant lesions met the inclusion criteria.

Exclusion criteria were neurological conditions influencing the esophageal motility, anatomical abnormalities of the oropharynx or local esophagus, mechanical obstruction, previous gastrointestinal surgery (gastrectomy, esophagectomy, esophageal myotomy, or fundoplication) or endoscopic procedure (esophageal dilatation), and history of drug intake impacting gastrointestinal (GI) motility.

The following factors were assessed: sex, the primary complaint for which conventional esophageal manometry was recommended, comorbidities, and drug history.

Esophageal pressure was measured as a part of the conventional manometry protocol at the LES level. The patient was instructed to swallow 5 ml of water (wet swallow) ten times with a 30-second gap between each time throughout the motility process. A conventional four-channel manometry catheter was used to assess the pressure and relaxation of the LES and the contractions of the esophageal body. The manometric tool utilized was polygraf; Synectics Medtronic.

The pressure sensors recorded and displayed data by transmitting intraluminal pressure signals to a receiving device.

The manometric analysis yielded the following results. The LES station analysis indicated a resting pressure range of 15 to 40 mmHg during wet swallows. Additionally, the distal esophageal body motility analysis showed a mean amplitude ranging between 40 and 120 mmHg during wet swallows [15]. For the various motility diseases, conventional manometry definitions were utilized (Table 1).

**Table 1:** Conventional manometric definitions of different motility disorders

Achalasia:	
1. Absence of distal esophageal peristalsis, which was necessary for a definitive diagnosis	
2. Elevated lower esophageal sphincter (LES) pressure at rest, typically above 45 mmHg	
3. Incomplete LES relaxation, characterized by a residual pressure greater than 8 mmHg;	
4. Elevated baseline esophageal pressure.	
Diffuse esophageal spasm:	
1. Simultaneous contractions during more than 20% of wet swallows	
2. Intermittent normal peristalsis (necessary for diagnosis)	
3. Repetitive contractions with more than three peaks;	
4. Prolonged duration contractions lasting more than 6 seconds with retrograde contractions.	
5. Isolated incomplete LES relaxation with residual pressure greater than 8 mmHg	
Nutcracker esophagus:	
1. Increased distal peristaltic amplitude exceeding 180 mmHg	
2. Increased distal peristaltic duration exceeding 6 seconds	
Scleroderma esophagus:	
1. Decreased LES pressure	
2. Weak or absent distal peristalsis	
3. Normal upper esophagus and upper esophageal sphincter	

All patients in group B underwent thorough geriatric evaluations, including cognitive (Mini-Cog score), functional, and clinical frailty scale (CFS) evaluations. The Mini-Clock Cog’s Drawing Test (CDT) section enables clinicians to quickly evaluate various cognitive functions, including memory, language comprehension, visual-motor skills, and executive function. Additionally, it provides a clear record of both healthy and abnormal performance that can be followed over time [16].

The MNA® evaluates nutritional status as a part of the routine examination of older patients in clinics, nursing homes, hospitals, or among those who are otherwise weak. A single, quick nutritional assessment tool was created [17]. The Katz Index of Independence in Activities of Daily Living, also known as the Katz ADL, is considered the best tool for evaluating functional status to measure a client’s ability to carry out daily activities independently [18].

The sample size was calculated assuming that the proportion of esophageal motility dysfunction in symptomatic patients is 50%. A minimum sample size of 385 individuals was required, with a margin of error of 0.05 and a 95% confidence interval. The sample size was estimated using NQuery statistical package, version 7.0, Los Angeles, CA.

This study was performed according to the ethical principles of the Declaration of Helsinki for good clinical practice. It was approved by the Ethical Research committee of the Faculty of Medicine, Cairo University (MD-204-2019). The possible side effects and complications associated with the procedure were communicated to all patients. All 385 patients signed a written informed consent that included their contact information.

### Statistical analysis

The numerical data were represented as mean ± SD, whereas the categorical data were reported as numbers and percentages. Student’s t-test and χ^2^-test were used when needed. Survival curves were plotted by the Kaplan-Meier method and compared using the log-rank test. Statistically significant differences were considered if the *P*-values were < 0.05.

## Results

The study included 385 patients, divided into two groups based on their ages. Group A included patients under 65, while Group B included patients over 65. The mean ages were 39 and 70 years, respectively. A total of 128 (47.1%) patients in group A and 63 (55.8%) in group B were females. Nutritional assessment using Mini Nutritional Assessment (MNA) short form score revealed that 10 patients (3.7%) in group A and 8 (7.1%) in group B were malnourished with a statistically significant difference (*P* = 0.003) as shown in Table 2.

**Table 2:** Demographic characteristics and nutritional assessment

	Group A	Group B	*P* value
	***n*** = 272 (%)^**a**^	***n*** = 113 (%)^**a**^	
Gender			
• Female	128 (47.1)	63 (55.8)	0.146
• Male	144 (52.9)	50 (44.2)	
Symptoms			
• Heartburn	81 (29.8)	36 (31.9)	0.716
• Reflux	124 (45.6)	49 (43.4)	0.736
• Dysphagia	101 (37.1)	55 (48.7)	0.040
• Odynophagia	13 (4.8)	9 (8)	0.233
• Dyspepsia	25 (9.2)	15 (13.3)	0.271
• Epigastric pain	38 (14)	22 (19.5)	0.216
• Atypical chest pain	42 (15.4)	15 (13.3)	0.639
MNA			
• Normal	202 (74.3)	64 (56.6)	0.003
• At risk	60 (22.1)	41 (36.3)	
• Malnourished	10 (3.7)	8 (7.1)	

*P* value < 0.05 is considered significant, ^a^Percentages were calculated within rows

The manometry examination revealed that the resting LES pressure was significantly lower in group A (40.8%) than in Group B (41.6%) (*P* = 0.001). In contrast, complete LES relaxation percentage and normal esophageal body peristalsis were significantly higher in Group A than in Group B (72.8% vs. 60.2%, respectively, *P* = 0.001) and (71.3% vs. 56.6%, respectively, *P* = 0.008). Approximately one-third of patients (33%) were diagnosed with achalasia, in which manometric results were significantly higher in Group B (43.4%) than in Group A (28.7%) (*P* = 0.016) (Table 3).

**Table 3:** Manometric study values

	Group A	Group B	*P* value
	Median (range)	Median (range)	
• LES resting pressure value	16 (3-91)	18 (5-70)	0.029
• LES relaxation percentage	100 (14-100)	100 (22-100)	0.364
LES resting pressure	**n (%)**^**a**^	**n (%)**^**a**^	
• Low	118 (43.4)	32 (28.3)	0.001
• Normal	111 (40.8)	47 (41.6)	
• High	24 (8.8)	13 (11.5)	
LES relaxation percentage	**n (%)**^**a**^	**n (%)**^**a**^	
• Incomplete	55 (20.2)	23 (20.4)	0.001
• Complete	198 (72.8)	68 (60.2)	
Esophageal body peristalsis	**n (%)**^**a**^	**n (%)**^**a**^	
• Normal	194 (71.3)	64 (56.6)	0.008
• Very weak	3 (1.1)	0 (0)	
• Simultaneous	70 (25.7)	49 (43.4)	
• Normal and simultaneous	3 (1.1)	0 (0)	
• Simultaneous and very weak	2 (0.7)	0 (0)	
Motility disorders			
Normal	72 (26.5)	27 (23.9)	0.016
Achalasia	78 (28.7)	49 (43.4)	
Motility disorders	122 (44.9)	37 (32.7)	
		No (%)	
• Normal		94 (24.4)	
• Achalasia		127 (**33**)	
• Hiatus hernia		4 (1)	
• Nutcraker esophagus		9 (2.3)	
• Weak esophageal body peristalsis		3 (0.8)	
• Low LES resting pressure		147 (38.2)	
• High LES resting pressure		1 (0.3)	

*LES* lower oesophageal sphincter, *P* value < 0.05 is considered significant, ^a^Percentages were calculated within rows

As shown in Table 4, the associations between motility disorders and heartburn, reflux, dysphagia, dyspepsia, and epigastric pain were statistically significant. Dysphagia was significantly higher in patients with evidence of achalasia. In contrast, heartburn, reflux, dyspepsia, and epigastric pain were significantly higher in patients with other motility disorders (*P* < 0.001).

**Table 4:** Relation between patient’s symptoms and different motility disorders

	Whole study population (*n* = 385)			*P* value
	Normal	Achalasia	Other disorders	
	***n*** = 99 (%)^**a**^	***n*** = 127 (%)^**a**^	***n*** = 159 (%)^**a**^	
Heartburn	38 (32.5)	14 (12)	65 (55.6)	< 0.001
Reflux	59 (34.1)	20 (11.6)	94 (54.3)	< 0.001
Dysphagia	22 (14.1)	109 (69.9)	25 (16)	< 0.001
Odynophagia	6 (27.3)	3 (13.6)	13 (59.1)	0.114
Dyspepsia	11 (27.5)	5 (12.5)	24 (60)	0.008
Epigastric pain	22 (36.7)	11 (18.3)	27 (45)	0.016
Atypical chest pain	13 (22.8)	14 (24.6)	30 (52.6)	0.152

^a^Percentages were calculated within rows

**Fig. 1:**
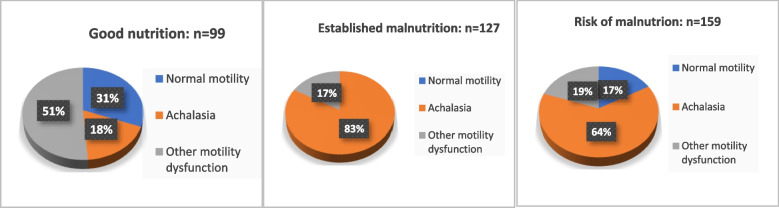
Relation between nutritional assessment and motility disorders

Compared to other motility disorders, patients with evidence of achalasia in the manometric study had a significantly higher risk of malnutrition (63.4% vs. 19.8%, *P* < 0.001) and established malnutrition (83.3% vs. 16.7%, *P* < 0.001) (Fig. 1).

Geriatric assessment of group B patients included cognitive assessment using the mini-cog score (attached) revealed that none of them had cognitive impairment. However, significantly higher frailty scores (4-7) were observed in achalasia patients compared to other motility disorders (58.8% vs. 27.9%, *P* = 0.016). Regarding functional status assessment using the Katz Index of Independence in Activities of Daily Living, Most achalasia patients (85%) in group B were more dependent than patients with other motility disorders (10%) (*P* = < 0.001) (Table 5).

**Table 5:** Relationship between geriatric assessment and motility disorders in geriatric group (group B)

	Normal	Achalasia	Other dysfunction	
	***n*** = 27 (%)^**a**^	***n*** = 49 (%)^**a**^	***n*** = 37 (%)^**a**^	***P*** value
Frailty				
• Very fit (1)	8 (40)	2 (10)	10 (50)	NA
• Well (2)	4 (33.3)	3 (25)	5 (41.7)	
• Managing well (3)	6 (46.2)	4 (30.8)	3 (23.1)	
• Vulnerable (4)	1 (8.3)	8 (66.7)	3 (25)	
• Mildly frail (5)	4 (16)	10 (40)	11 (44)	
• Moderately frail (6)	3 (16.7)	11 (61.1)	4 (22.2)	
• Severely frail (7)	1 (7.7)	11 (84.6)	1 (7.7)	
Frailty groups				
• Healthy (1-3)	18 (40)	9 (20)	18 (40)	< 0.001
• Frail (4-7)	9 (13.2)	40 (58.8)	19 (27.9)	
ADL				
• Independent	18 (33.3)	15 (27.8)	21 (38.9)	< 0.001
• Intermediate	8 (20.5)	17 (43.6)	14 (35.9)	
• Severe impairment	1 (5)	17 (85)	2 (10)	

^a^Percentages were calculated within rows

## Discussion

Dysphagia is a warning sign that needs to be evaluated immediately to pinpoint the specific cause and start the right course of treatment. It might be brought on by an irregularity in the structure or motility of the transit of solids or liquids from the mouth to the stomach.

Since the 1960s, numerous studies have sought to show how aging affects esophageal motility, with various results [2]. However, dysphagia in older persons should not be attributed to normal aging because aging alone generates modest esophageal motility abnormalities, which are rarely symptomatic [19]. After ruling out mechanical esophageal obstructions, esophageal manometry study (EMS) became a crucial diagnostic tool in analyzing patients with chest discomfort and dysphagia of esophageal origin. The statistics suggest a contentious relationship between the patient’s complaints and the motility problems identified by the esophageal manometric examination. Additionally, it confirms the impact of age on esophageal motility disorders and suggests a possible link between gender and several motility diseases.

In addition, our findings demonstrated a robust association between esophageal motility abnormalities and geriatric and nutritional assessment. They showed a contentious relationship between certain upper GI symptoms and conventional esophageal manometry studies. Dysphagia was significantly higher in patients with achalasia, while heartburn, reflux, dyspepsia, and epigastric pain were higher in patients with other motility disorders. On the other hand, some researchers found no association between esophageal symptoms and manometric evidence of motility abnormalities. This was partially attributed to the high administration rate of proton pump inhibitors (PPIs) and other medications that can potentially interfere with manometry tests [20].

Achalasia was diagnosed in 33% of patients in the current study, this high incidence is in agreement with the study published by Rehman H. et, al who found that 72 out of 202 patients (35.6%) presenting with dysphagia were diagnosed with achalasia [21]. This disagree with Canadian incidence of achalasia (1.63/100,000), this variation may be attributable to case discovery, where only subjects attending for treatment by pneumatic dilatation or surgical myotomy were included [22]..

The current study demonstrated that nearly half (48.7%) of patients in Group B had a history of dysphagia. Additionally, Group B patients had a higher prevalence of achalasia. Multivariate analysis demonstrated a two-fold increase in the risk of achalasia in Group B (age ≥ 65 years) compared to group A (age ≤ 65 years),. Additionally, the increased loss of esophageal myenteric plexus neurons in the smooth muscle layers of the esophageal wall causes alterations in esophageal physiology in the elderly population [23].

According to several studies, there is a statistically significant association between esophageal motility and age. While some of these studies involved healthy participants, others were symptomatic patients [24]. In contrast, other studies refuted this association between esophageal motility and age and declared the absence of any significant difference in LES pressure based on age [25].

The nutritional requirements of adults are impacted by various factors, including their activity level, energy expenditure, and caloric needs. Additionally, their access to food, ability to prepare and ingest food, food preferences, specific health issues, and compromised organ systems must be considered. Further, homeostatic regulation is weakened with aging, leading to a decline in organ system reserves. Up to 71% of older hospitalized persons may be nutritionally vulnerable or malnourished [26].

Another study by Sweed et al. revealed that among 200 Egyptian participants residing in senior care facilities, 8.5% were suffering from malnutrition, 48% were at risk of malnourishment, and 43.5% were in good nutritional condition [27].

Esophageal motility abnormalities impact the patient’s nutritional condition. Our research aimed to explore and support this theory by utilizing the MNA score to evaluate nutritional status. Patients with achalasia had a greater prevalence of malnutrition risk and actual malnutrition. Similar results were reported in a study that examined the nutritional status of 171 patients with achalasia using the malnutrition universal screening test (MUST score). It revealed that over 70% had a moderate to high risk of malnutrition [28].

Geriatric evaluation of the elderly patients (Group B) demonstrated that achalasia patients had a higher risk of functional impairment (evaluated by ADL index) and frailty (measured by frailty score) than those with normal esophageal motility or other motility disorders. The higher likelihood of malnutrition among achalasia patients may help explain this finding. According to the MNA score, 65% of frail people in a study of 112 elderly subjects were at risk of malnutrition [29].

A limitation of this study is that uncontrolled diabetes, poor dentation, and drugs, such as anticholinergics and antihistamines that hinder food bolus transport in the elderly, are potential causes of dysphagia that may interfere with the manometric findings. Another study limitation was the application of conventional manometry instead of the higher-resolution manometry, which was unavailable in our unit. Despite being more expensive, the latter approach has the advantage of measuring the upper and lower esophageal sphincter pressure and the esophageal body peristalsis simultaneously with a single series of swallows, reducing the time needed for data gathering.

In conclusion, Achalasia is an important cause of dysphagia in elderly patients, predisposing them to malnutrition and functional impairment. When caring for the elderly, a multidisciplinary approach is essential, paying close attention to polypharmacy and the adverse effects of the drugs affecting esophageal motility.

## Supplementary Information

**Additional file 1.**

## Data Availability

All data generated or analyzed during this study are included in this published article.
