# Supplementary material for: Esophageal motility disorders in symptomatic patients and its relation to age
Source: BMC Gastroenterol. 2023 Mar 11;23:69. doi: 10.1186/s12876-023-02709-3 (PMC10007782; doi:10.1186/s12876-023-02709-3)
Supplement: Supplementary file 1 — Additional file 1. [file 12876_2023_2709_MOESM1_ESM.docx]

Scoring system of Mini-Cog

| **ADMINISTRATION** | | **SPECIAL INSTRUCTIONS** |
| --- | --- | --- |
| **1** | **Get patient’s attention and ask him or**  **her to remember three unrelated words. Ask patient to repeat the words to ensure the learning was correct.** | - **Allow patient three tries, then go to next item.** - **The following word lists have been validated in a clinical study:**(215–217)   **VERSION 1 VERSION 3 VERSION 5**   - - **Banana • Village • Captain**   - **Sunrise • Kitchen • Garden**   - **Chair • Baby • Picture**   **VERSION 2 VERSION 4 VERSION 6**   - - **Daughter • River • Leader**   - **Heaven • Nation • Season**   - **Mountain • Finger • Table** |
| **2** | **Ask patient to draw the face of a clock.**  **After numbers are on the face, ask patient to draw hands to read 10 minutes after 11:00 (or 20 minutes after 8:00).** | - **Either a blank piece of paper or a preprinted circle (other side) may be used.** - **A correct response is all numbers placed in approximately the correct positions AND the hands pointing to the 11 and 2 (or the 4 and 8).** - **These two specific times are more sensitive than others.** - **A clock should not be visible to the patient during this task.** - **Refusal to draw a clock is scored abnormal.** - **Move to next step if clock not complete within three minutes.** |
| **3** | **Ask the patient to recall the three words from Step 1.** | **Ask the patient to recall the three words you stated in Step 1.** |

| **SCORING** | |
| --- | --- |
| **3 recalled words** | **Negative for cognitive impairment** |
| **1-2 recalled words + normal CDT** | **Negative for cognitive impairment** |
| **1-2 recalled words + abnormal CDT** | **Positive for cognitive impairment** |
| **0 recalled words** | **Positive for cognitive impairment** |

Mini Nutritional Assessment

**MNA^®^**


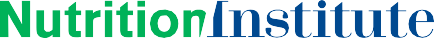


| Screening |
| --- |
| **A Has food intake declined over the past 3 months due to loss of appetite, digestive problems, chewing or swallowing difficulties?**  0 = severe decrease in food intake  1 = moderate decrease in food intake  2 = no decrease in food intake  |
| **B Weight loss during the last 3 months**  0 = weight loss greater than 3 kg  1 = does not know  2 = weight loss between 1 and 3 kg  3 = no weight loss  |
| **C Mobility**  0 = bed or chair bound  1 = able to get out of bed / chair but does not go out  2 = goes out  |
| **D Has suffered psychological stress or acute disease in the past 3 months?**  0 = yes 2 = no  |
| **E Neuropsychological problems**  0 = severe dementia or depression 1 = mild dementia  2 = no psychological problems  |
| **F1 Body Mass Index (BMI) (weight in kg) / (height in m)^2^**  0 = BMI less than 19  1 = BMI 19 to less than 21  2 = BMI 21 to less than 23  3 = BMI 23 or greater  |
| IF BMI IS NOT AVAILABLE, REPLACE QUESTION F1 WITH QUESTION F2. DO NOT ANSWER QUESTION F2 IF QUESTION F1 IS ALREADY COMPLETED |
| **F2 Calf circumference (CC) in cm**  0 = CC less than 31  3 = CC 31 or greater 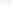 |

Clinical Frailty Scale

| **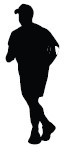** | **1. Very Fit - People who are robust, active, energetic and motivated. These people commonly exercise regularly. They are among the fittest for their age.** |
| --- | --- |
| **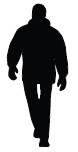** | **2. Well - People who have no active disease symptoms but are less fit than category 1. Often, they exercise or are very active occasionally, e.g'. seasonally. Well older adults share most attributes of the very fit, except for regular, vigorous exercise. Like them some may complain of memory symptoms, but without objective deficits.** |
| **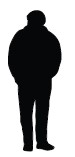** | **3. Managing Well - People whose medical problems are well controlled, but are not regularly active beyond routine walking. Those with treated medical problems who exercise are classed in categories 1 or 2.** |
| **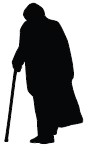** | **4. Vulnerable - While not dependent on others for daily help, often symptoms limit activities. A common complaint is being "slowed up", and/or being tired during the day. Many rate their health as no better than "fair". Memory problems, if present, can begin to impact on function (e.g. having to look up familiar recipes, misplacing documents) but usually do not meet dementia criteria. Families often note some withdrawal - e.g. needing encouragement to go to social activities.** |
| **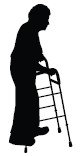** | **5. Mildly Frail - These people often have more evident slowing, and need help in high order IADLs (finances, transporiation, heavy housework, medications). Typically, mild frailty progressively impairs shopping and walking outside alone, meal preparation and housework. Often, these people have several illnesses, and take several medications. This category includes people with mild dementia. Their common symptoms include forgetting the details of a recent event, even though they remember the event itself, asking the same question, or telling the same story several times a day and social withdrawal.** |
| **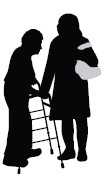** | **6. Moderately Frail - People need help with all outside activities and with keeping house. Inside, they often have problems with stairs and need help with bathing and might need minimal assistance (cuing, standby) with dressing. If a memory problem causes the dependency o often recent memory will be very impaired o even though they seemingly can remember their past life events well.** |
| **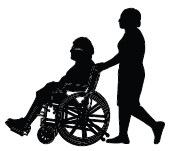** | **7. Severely Frail - Completely dependent for personal care, from whatever cause (physical or cognitive). Even so, they seem stable and not at high risk of dying (within - 6 months).** |
| **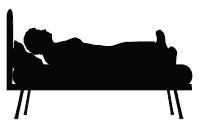** | **8. Very Severely Frail - Completely dependent, approaching the end of life. Typically, they could not recover even from a minor illness.** |
| **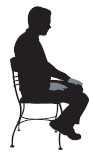** | **9. Terminally Ill - Approaching the end of life. This category applies to people with a life expectancy of <6 months, who are not otherwise evidently frail** (221)**.** |

Katz Index of Independence in Activities of Daily Living (ADL)

| **Katz Index of Independence in Activities of Daily Living** | | |
| --- | --- | --- |
| **Activities**  Points (1 or 0) | **Independence**  (1 Point)  **NO** supervision, direction or personal assistance. | **Dependence**  (0 Points)  **WITH** supervision, direction, personal assistance or total care. |
| **BATHING**  Points: | **(1 POINT)** Bathes self completely or needs help in bathing only a single part of the body such as the back, genital area or disabled extremity. | **(0 POINTS)** Need help with bathing more than one part of the body, getting in or out of the tub or shower. Requires total bathing |
| **DRESSING**  Points: | **(1 POINT)** Get clothes from closets and drawers and puts on clothes and outer garments complete with fasteners. May have help tying shoes. | **(0 POINTS)** Needs help with dressing self or needs to be completely dressed. |
| **TOILETING**  Points: | **(1 POINT)** Goes to toilet, gets on and off, arranges clothes, cleans genital area without help. | **(0 POINTS)** Needs help transferring to the toilet, cleaning self or uses bedpan or commode. |
| **TRANSFERRING**  Points: | **(1 POINT)** Moves in and out of bed or chair unassisted. Mechanical transfer aids are acceptable | **(0 POINTS)** Needs help in moving from bed to chair or requires a complete transfer. |
| **CONTINENCE**  Points: | **(1 POINT)** Exercises complete self control over urination and defecation. | **(0 POINTS)** Is partially or totally incontinent of bowel or bladder |
| **FEEDING**  Points: | **(1 POINT)** Gets food from plate into mouth without help. Preparation of food may be done by another person. | **(0 POINTS)** Needs partial or total help with feeding or requires parenteral feeding. |
| **TOTAL POINTS: SCORING:** 6 = High (*patient independent*) 0 = Low (*patient very dependent* | | |
